# Bats, bugs, and babies: why stringent guidelines are needed for cause-and-effect interpretation in epidemiological studies

**DOI:** 10.1007/s00204-025-04066-4

**Published:** 2025-06-18

**Authors:** Philip Marx-Stoelting, Tewes Tralau, Veronika Städele, Stefanie Rotter, Vera Ritz, Jörg Rahnenführer, Jan G. Hengstler

**Affiliations:** 1 Department of Pesticides Safety, German Federal Institute for Risk Assessment (BfR), Berlin, Germany; 2 Department of Statistics, TU Dortmund University, Vogelpothsweg 87, 44227 Dortmund, Germany; 3 Department of Toxicology, Leibniz Research Centre for Working Environment and Human Factors (IfADo), Dortmund, Germany

## Abstract

In a recently published study (Frank, 2024), the author claimed that farmers in the USA compensated for bat decline by increasing their insecticide use by 31.1%, which allegedly caused a 7.9% increase in human infant mortality. The author concluded that his “result highlights that real-world use levels of insecticides have a detrimental impact on health, even when used within regulatory limits”. The study is a prime example of how statistics should not be used in a design based on a classical logical fallacy. That is, in short, factor A (detection of a lethal bat disease) correlates with factor B (increased insecticide use) and factor A also correlates with factor C (increased infant mortality). Based on a flawed logic, the author concludes that consequently a causal relationship exists between factor B (pesticide use) and factor C (infant mortality). Remarkably, the causal relationship between increased pesticide use (factor B) and increased infant mortality (factor C) was claimed despite not investigating a statistical correlation between these two factors. The study also contains numerous important but less obvious flaws, including the use of aggregated data to make inferences about individual outcomes; inadequate consideration of maternal characteristics; a suboptimal proxy for bat population declines; inadequate timing of ‘treatment’; assumption of quasi-randomness; questionable binning of coefficients and a lack of data documentation. Scientifically, the article highlights the need for more stringent statistical and scientific quality controls. However, given the implications such cavalier claims have in the context of public health, it also shows that the scientific community urgently needs a better understanding of the robustness of statistical conclusions in the context of epidemiological and ecological studies.

## The claim of causality is not justified

The study of Frank (2024) postulates causal relationship between bat decline due to white-nose syndrome (WNS) leading to increased use of insecticides and, consequently, to increased infant mortality. Specifically, the author claims (abstract): “I find that farmers compensated for bat decline by increasing their insecticide use by 31.1%. The compensatory increase in insecticide use by farmers adversely affected health—human infant mortality increased by 7.9% in the counties that experienced bat die-offs.” This assertion is repeated even more emphatically in the discussion: “I demonstrate how declines in insect-eating bat population levels induce farmers to substitute with insecticides, consequently resulting in a negative health shock to infant mortality.”

The claims as such are remarkable and, if substantiated, would indeed be worrisome—not least because they would imply a massive failure of toxicology and public healthcare and would do so in the context of one of the most comprehensively regulated areas of toxicology. Given these implications, it is noteworthy that neither the methods used to substantiate this claim, nor the study design are suitable to come to any of these conclusions.

**Fig. 1:**
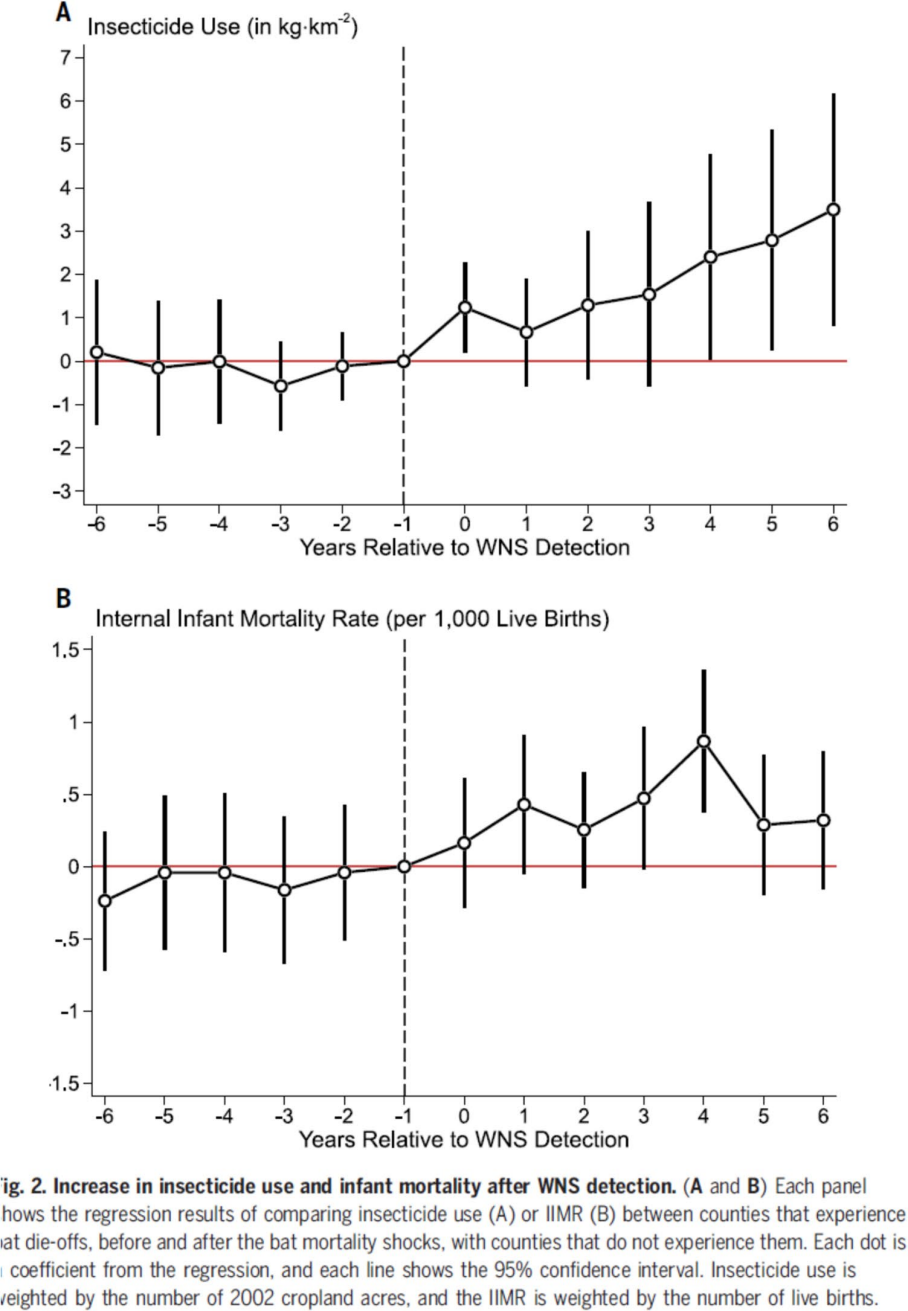
Reproduction of Fig. 2 of Frank (2024). The data were used to claim a causal relationship between increased insecticide use after detection of white-nose syndrome (WNS) and increased infant mortality. The data show that the 95% confidence intervals overlap the zero line for most years and should, therefore, not be considered as statistically significant. Statistical significance was only obtained after application of a binning procedure. It should also be noted that the coefficients increase continuously from year 4–6 for insecticide use, whereas the coefficients of infant mortality decrease from year 4 to 5

The reason is that the “difference in difference” (DiD) study is limited to the analysis of two associations, that is, (1) the association of WNS detection and insecticide use at the county level and (2) the association of WNS detection and infant mortality at the county level. What it does not analyze is the association between insecticide use and infant mortality, which is the very causal relationship that is claimed. This is important because association of variable A and B and additionally association of variable B and C do not necessarily imply an association of variables B and C, and certainly not a causal relationship between B and C. Moreover, the coefficient that quantifies the change in infant mortality increases up to year 4 after WNS detection but decreases again from year 4 to year 5, with the 95% confidence interval of the coefficient containing zero in years 5 and 6 (Fig. 2B of Frank 2024; reproduced in Fig. 1), as was also the case up to year 3. Contrastingly, the coefficient that quantifies the change of insecticide use continuously increases in the corresponding time period (years 4–6), over which the coefficient of infant mortality decreases (Fig. 2 A). Even in absence of any further statistical analysis, this already indicates that any claim of the coefficients of insecticide use and infant mortality being associated with each other should be questioned. Further data on infant mortality that would allow a more in-depth analysis of a possible association of insecticide use and infant mortality are unfortunately not provided. A further critical point is that the claim that human infant mortality increased by 7.9% is made without any statement about the statistical uncertainty of this number.

## The statistical problem of using aggregated data to predict individual outcomes

It should be noted that the study relies on data aggregated at the county level. This inherently bears the risk of a well-documented statistical phenomenon, namely the ‘ecological fallacy’. This term describes the fact that associations at the aggregate level do not necessarily mean that these very same associations also exist at the individual level (Robinson 1950, Wakefield 2008). This principle is illustrated by hypothetical numbers in Table 1, where variable A and B are almost perfectly correlated at the group level (aggregated data) but not at the individual level. Infant mortality is a complex outcome determined by many individual factors which comprise some intrinsic to the infant, as well as parental, economic and hereditary factors, health care, the immediate local environment, and so on. None of these can be accounted for using county-aggregate measures.

**Table 1:** Simple numerical example of the ecological fallacy. Variables A and B correlate with each other when means of each group are considered. However, variables A and B do not correlate at the individual level

Individual	Group	Variable A	Variable B	Group mean A	Group mean B
1	1	0	3		
2	1	1	0		
3	1	0	2		
4	1	2	0	0.75	1.25
5	2	1	4		
6	2	2	1		
7	2	1	3		
8	2	3	0	1.75	2
9	3	2	5		
10	3	3	2		
11	3	2	4		
12	3	4	1	2.75	3
13	4	3	6		
14	4	4	3		
15	4	3	5		
16	4	5	1	3.75	3.75
Correlation (A, B)				− 0.072	(− 0.55 to 0.44)
Correlation (mean A, mean B)				0.998	(0.87–1.00)

Pearson’s product moment correlation coefficient; 95% confidence intervals in parentheses

Even insecticide use could be considered an individual property as the level of insecticide use may be driven by factors unique to a particular farm. This includes needs of the specific crops, varying levels of local pest populations, as well as the individual experience of loss of income due to insect infestation and perceived risk of further losses. Other possible confounders are environmental pollution, consideration of drift of insecticides or shift of bat populations due to other factors, neither of which is restricted to county boundaries.

As a result of these limitations, caution should be exercised in any case where complex causal relationships occurring at individual levels are deducted from aggregated data. To be robust, the plausibility of any such claims, therefore, requires substantial additional testing and statistical analysis of data collected at the individual level.

## The possible influence of maternal characteristics on infant mortality is not adequately considered

The author claims to take into account differences between mothers (e.g., Supplement, p.18) as potential predictors of infant mortality by demonstrating that characteristics of mothers averaged at the county level do not change for counties after emergence of WNS.

The same DiD equation was used in all analyses with each maternal characteristic as a separate outcome (Supplement, p. 18). Importantly, confidence intervals around coefficients do not contain zero for some of the characteristics (Fig. S12); therefore, some of the maternal characteristics seem to differ statistically significantly before and after WNS detection. The differences are relatively small (max. ~ 4%) between mothers in WNS and non-WNS counties, leading the author to conclude that maternal characteristics do not play a relevant role in driving the observed effect of increased infant mortality. However, a relatively small effect of a confounder does not automatically mean that it is not relevant; rather the main analysis should have been adjusted for these confounders. Others have shown that DiD analysis can produce biased results when a time-varying covariate that predicts differential changes between the group with intervention and the reference group (here: with and without WNS detection) is not included as a confounder in the analysis (Zeldow & Hatfiled, 2021). Whether maternal characteristics are time-varying between WNS and non-WNS counties is not clear from the results presented in Fig. S2, as they show the average treatment effect for years after treatment.

It is also questionable whether the most relevant maternal factors affecting infant mortality were considered. The author used the data “in the birth certificates on the age category, educational attainment category, share of mothers who are white, married, the mean number of prenatal care visits, and the share of mothers that were smoking for at least some part of the pregnancy (Suppl. P. 18)”. It is not clear if this information is sufficient to control for the influence of maternal factors. For example, it has previously been shown that maternal income and minimal wage are associated with infant mortality (Wolf et al. 2021). It remains unclear how this important influential factor was considered in the present study (Frank 2024). As discussed above, such individual maternal factors cannot be addressed at the aggregated level (counties), as conducted by Frank (2024), but should be considered at the individual level.

## Confirmed detection of WNS is an inadequate proxy for reduction in local bat populations

It is unclear how suitable WNS detection is as a proxy for actual declines in the bat population. It is likely that counties are heterogeneous with regard to the spread of the disease, bat population size prior to appearance of the disease, bat habitat, overlap of bat ranges and agricultural land, and composition of the bat population, especially given that different species show different degrees of susceptibility (Mallinger et al. 2023). The author attempts to resolve this issue by utilizing two alternative measures: number of susceptible bat species and three measures of WNS severity including actual estimates of bat population decline (Supplementary, p.15).

However, the author himself states that the number of susceptible bat species might be a poor predictor of bat abundance. Moreover, the study relies on survey data from Cheng et al. (2021) for estimates of WNS severity. This reports the percent decline in bat populations, the number of affected sites, and the estimated number of bat losses. Notably, they do so only for a subset of counties. Repeating the analysis with actual population decline data, the author sees the hypothesized causal relationship between WNS and infant mortality substantiated. For example, a loss of 100,000 bats (median county loss) is estimated to increase the internal infant mortality rate (IMR) by 0.007 per 1000, while the estimated mean increase after detection of WNS is 0.54 deaths per 1000. It is subsequently stated that the results are to be interpreted with caution as population decline data are county means of surveys at individual sites that were not intended to obtain representative county means. However, this information is unfortunately not reflected in the further use of this data.

Further, the timing of ‘treatment’ is uncertain. In the present study, ‘treatment’ is the year of the confirmed infection of bats with the fungus causing WNS. The author identified empirically that there is a median delay of 2 years (0–4, Fig. S6) between a county being suspected of being WNS-positive and being confirmed WNS-positive and consequentially subtracted 2 years from all treatment years to adjust for this delay. This means, however, that there may be counties for which ‘treatment’ was assigned up to 2 years earlier than it actually occurred, and counties for which treatment was assigned up to 2 years later than it actually occurred. This uncertainty in the timing of infestation is contrary to the standard usage of DiD analyses, in which the precise timing of treatment (e.g., the passing of a law, implementation of a program) is known. By definition, DiD imposes an assumption of no treatment anticipation (treated units will not adjust their behavior prior to treatment in anticipation of treatment) and the uncertainty in the timing of treatment leads to a violation of this assumption.

The unclear beginning of “treatment” may critically influence the results. This can be illustrated by considering a theoretical case in which WNS detection in all counties was identified 2 years early by shifting the red line (representing the reference one year prior to WNS detection) in Fig. 2 A and B by 2 years from the point estimate for year − 1 to the point estimate for year + 1. In this case, the upward trend of the coefficients for insecticide use would be smaller and for infant mortality, no discernible effect over time would remain. Again, it should be considered that even in its present form, the 95% confidence intervals of the coefficients overlap zero for almost all years.

## The assumption of the quasi-randomness of the WNS expansion is not fulfilled with regard to spatial proximity

The author tests two assumptions of the analysis: (1) whether pesticide use or infant mortality in a previous year predict WNS status in the following year (Supplementary, p. 20). In this instance, previous pesticide use and infant mortality did not predict WNS status in the following year (Table S14). (2) Whether the WNS status of a county in a previous year predicts WNS status of a neighboring county in the following year (Supplementary, p. 20). This is indeed the case and having a neighboring county with WNS in the previous year results in an 84% probability of being classified as WSN county the following year (Table S14). Therefore, quasi-randomness of the WNS expansion with regard to spatial proximity, one of the fundamental assumptions of DiD, is not given.

## Binning of event-time coefficients

The event-time analysis presented in Fig. 2 by Frank (2024) shows that the 95% confidence intervals around the coefficients from the regression of infant mortality on WNS status overlap zero for most of the years analyzed, even after the onset of WNS detection, suggesting that confidence in the effects being different from zero is low. Indeed, formal statistical significance is obtained only once event-time coefficients are binned into 2-year intervals (results shown in Table S3). To justify this procedure, the author cites two publications (Miller 2023; Borusyak, 2024), one of which points out potential bias due to such binning. It remains unclear whether binned data have also been used to generate results shown in Fig. 3 of Frank (2024). In conclusion, the requirement of binning to gain statistical significance, in combination with the large uncertainty in the effect, suggests that (a) the null hypothesis of no association between WNS status and infant mortality should not be rejected prematurely and (b) the moderate changes of the coefficients may simply be explained as fluctuations in zero noise.

## Lack of data documentation

A high-quality study should include a documentation of data that would allow the reader to replicate the findings. However, infant mortality data—key to the present study—are not provided and the reason given is data protection. While it remains difficult to understand why county-wise data on infant mortality should be so sensitive that publication must be prohibited, the authors of this commentary acknowledge that this was not necessarily under the author’s control. Nevertheless, the lack of the availability of key data strongly reduces the value of the study, since replication of the results is not possible.

## Conclusion

The claim made by Frank (Frank 2024) of causality between insecticide use and infant mortality is not justified. In particular, the use of data aggregated at the county level is inadequate to support the proposed causal relationships. It should be considered that human biomonitoring of insecticide exposure would allow an analysis at the individual level. Similarly, close monitoring of local bat populations and surveying of insecticide use by local farmers is possible. Therefore, while certainly an elaborate endeavor, adequate data to more convincingly investigate the hypotheses (causal relationships exist between bat population decline, pesticide use, human exposure and infant mortality) could be collected. While the increasing global threat to ecosystems certainly creates a need for identifying which ecosystem services are most crucial to human health, Frank (2024) presents little convincing evidence that they have found an important piece to the puzzle. More importantly, it starkly highlights the pitfalls of making health claims based on inadequate statistical and epidemiological analyses. Given the importance of the topic and the potential scientific and societal implications resulting from such claims, this example of a poor evaluation highlights the need for more rigorous scientific and statistical quality checks. 
